# What we see and what we do not see in health care

**DOI:** 10.3325/cmj.2011.52.215

**Published:** 2011-04

**Authors:** Nataša Škaričić

Fourteen years ago, US Inspector General and the Department of Health and Human Services conduced a detailed analysis of money outflow from the Medicare and Medicaid federal health care programs; their findings shocked the nation: US $23 billion was lost annually to different forms of fraud and corruption, reaching 14% of the total health budget for both federal programs (1). This criminal activity, formally termed ‘white-collar fraud,’ was carried out through spectacular operation of falsifying and ‘building up’ of invoices, opening of false firms, insurance fraud, charging non-existent laboratory and other tests, and other fairly complicated but extremely lucrative criminal ventures. Only a year after the first report, the amount lost to health care programs dropped to US $20.3 billion and to US $12.6 billion after two years (1), which can be ascribed to continuing analysis and money flow control, as well as detection and processing of fraud cases. Malcom Sparrow’s book License to Steal provides an analysis of the ways used to bleed money from the health care system and reveals a lucid truth: “What you see is not a problem. What you don’t see creates a serious damage to the system, and the effectiveness of the control mechanisms is measured by how many invisible problems they discover and prevent.” Sparrow detected another appalling phenomenon in health care – that many criminal groups, until recently concentrated on drug smuggling and other serious criminal deeds, have been redirecting their activities to health care fraud for two simple reasons – because it brings enormous monetary return and because the punishment for the acts, if they are discovered, are much more lenient than in ‘conventional’ street or organized crime. Sparrow also states that, because the types of fraud employed are ingenious, an important task for the health care management is to develop a good control system so that the theft from the taxpayers and damage to the quality of the health care are reduced.

Fraud and corruption in the public health care is recognized at the global level and is perhaps the biggest contemporary challenge to the financial management in health care. I write this in regard to the Medikol scandal that has been raging in Croatia, and has been branded by the media as “the biggest affair of Croatian health care.”

The essence of the affair, about which I have written a lot for the Croatian media (2,3) is the following:

1. A private entrepreneur (caterer) and his firm Medicol sign the first contract with the Croatian Institute of Health Insurance (CIHI) for the compensation for PET/CT diagnostic examinations, which he installed at the premises rented from one of the hospitals in Zagreb, Sisters of Mercy University Hospital Center. The contract is lucrative and favorable for the private business, as Medikol charges HRK 9000 (€ 1220, US $1800) for each of the contracted 4500 patients (total HRK 40.5 million). The previous cost of PET/CT scans for Croatian patients paid to institutions outside of Croatia by the CIHI was about HRK 10 million annually. A sudden jump in the system’s spending after the introduction of a private interest is obvious.

2. In the same year, Medikol sends the CIHI an outline of a ‘PET/CT Project for Croatia,’ which announces its business growth (6 new centers), defines the price of the service (€ 1300), and predicts a 25% annual increase in services charged to the CIHI.

3. Although there is no answer to this proposal in the archives of the CIHI or the Ministry of Health, the other documents (correspondence between the CIHI and the state hospitals in 2009) witness that the CIHI “abides by the agreement between the Ministry and Medikol about Medikol’s PET/CT Project in Croatia, which is carried out by Medikol.” In other words, the government financially supported the monopoly of a private businessman to PET/CT scan services. In 2009, Rijeka University Hospital Center, Jordanovac Hospital for Lung Diseases, Zagreb, and Osijek University Hospital each request a purchase of a PET/CT machine from the Ministry, but are all refused with the explanation that Medikol is the only institution that can have this machine with services contracted with the CIHI. This violates the Croatian regulations and is evidently a corrupt relationship with a private firm that made more than HRK 150 million profits in only four years.

4. After these facts were published in the media, we learn that the Croatian Office for Corruption and Organized Crime (USKOK) is investigating the case, and that two ministers of health, Neven Ljubičić and Darko Milinović, claim that the order to sign the contract came from the former prime minister Ivo Sanader (3), who has been arrested and is now in jail in Austria. Former minister Ljubičić signed the first contract with Medikol in 2007, and current minister Milinović has continued with the implementation of Medikol’s ‘Project PET/CT’ since 2008.

These are the facts that Sparrow would call “that what can be seen right away,” but are they the real heart of this scandal? Where is that that cannot be seen – what is it that we have to think and really worry about?

The main issue is that the USKOK’s action and media turmoil come three years after Medikol made the first agreement with the CIHI. This tells us that Croatia not only lacks any analysis of health care budget spending or precautionary measures to prevent irrational and questionable financial outflow, but that the functioning of the legal system is inhibited and the system does not react when an irregularity or criminal offense is noticed. Any anticorruption activity is missing, either before or after the discovery of corruption.

Let’s put aside that this conclusion is a part of general public attitude about the institutions in health care and legal system: when somebody tries to investigate an allegation of corruption or establish the political responsibility of the main actors, there are no leaders at the head of the health care or legal system to whom they could turn to, although this is the responsibility of all state institutions whose job is the prevention of corruption. Not to mention that there are many situations similar to Medikol. Let’s list just some of them.

Since 1999, the Ministry of Education and Sports has been purchasing the best magnetic resonance imaging (MRI) machines for the Neuron Polyclinic, public daughter company of the University of Zagreb School of Medicine, which performs commercial MRI scans. Nobody has ever made an inspection to whom and how the earnings from these expensive scans are distributed because Neuron is registered as a non-profit public organization. The institutions of the legal system have remained passive even after they learned in 2007 that the CIHI pays HRK 2 million annually for the scans performed in Neuron (4,5).

Furthermore, the Croatian state has been sending pediatric cardiology patients for surgeries to a private clinic in Linz (6), Austria, for eight years, and nobody has questioned the reasons why we would not do such surgical procedures ourselves when we have many experts, and why these services are persistently contracted to a single private institution.

Another example: the public (and the tax payers) do not know anything about 356 private health institutions that signed contracts with the CIHI in the last two years (7), during which the state health care institutions have experienced serious budget cuts because of rationalization and budget savings. Which are those private institutions? What are the reasons for directing budget funds to them while budget is cut for public institutions? Is there an explanation at all? What are the funds contracted? Is there any control of how they are spent?

More questions: what is the principle for making contracts with private institutions for home care and physical therapy (8)? Can anyone enter this network once it is closed or is there a monopoly established? Why are services contracted for ten years?

Why do imaging machines in the public hospitals work with 30% of their capacities while patients wait for 6 months for scans and the state sends them to private institutions (9)?

How come that the greatest number of public-private contracts is made at hemodialysis departments and why is hemodialysis perceived as a lucrative private initiative in health care (9)?

These and many others are all well-known cases of financial outflow from the mandatory solidarity health care insurance to the pockets of private businesses – the fact that we do not follow the money trail does not mean that were are not fully aware of the situation. What is needed is that the responsible legal body, such as the Office for Corruption and Organized Crime, takes a look into the business relationships between the state and the private sector.

What we can see even without legal investigation is that there is a fundamental difference between the US and Croatian experiences with the corruption in the health care system: the US system fights against the ingenuity of the private criminal firms that find ways to cheat the federal insurance, while in Croatia it is the administration that dictates the corruptive actions and allows selected private businesses to work under irrationally favorable conditions. In other words, the Croatian public health care system consciously services private businesses by providing a normative and procedural framework for them.

This brings us back to the story about Medikol: some media suggested that the Croatian health care would not be helped if this case was opened and the corruption revealed. That this is not true is clear already from the fact that the leading people from medical circles who today publicly denounce the Medikol case have been formally or informally in charge of the Croatian health care system and have contributed to its failure. Also, the whole case is now reduced to individuals and their roles, political intrigue and financial manipulation, while the structure of the health care system and the role of the whole social environment that made this case possible are not addressed. The main social framework was that of changing the social contract for health care, training the public to believe in the efficient functioning of the private and public health sector, and the poverty of the latter, so that patients are happy when some private businessman ‘leans on’ the public budget and don’t ask questions about their tax money. Moreover, this county has still not had a government or a minister of health, or any other supervising body, who would want to assess the current mechanisms to prevent budget losses from the public health sector, regardless of the health politics or political option in power.

We are so far away from this that we come again to the same conclusion – it is not businessmen who cheat the government, but the government deceives its insurers, impoverishes the public sector, and puts money in the hands of the private businesses.

Unfortunately, it seems that it is the plan of all future health policies to legalize such practice, ie, to support the private health entrepreneurship at the expense of the public sector by providing them the way out of the gray zone of corruption. In this respect, the Medikol case should remind us that it is the high time for all stakeholders in health care to try to establish some control over health care processes because the consequences of the lack of control are atrocious and deeply unjust.
